# Larval Breeding Sites of *Lutzomyia longipalpis* (Diptera: Psychodidae) in Visceral Leishmaniasis Endemic Urban Areas in Southeastern Brazil

**DOI:** 10.1371/journal.pntd.0002443

**Published:** 2013-09-19

**Authors:** Cláudio Casanova, Maria T. M. Andrighetti, Susy M. P. Sampaio, Maria L. G. Marcoris, Fernanda E. Colla-Jacques, Ângelo P. Prado

**Affiliations:** 1 Superintendência de Controle de Endemias, Secretaria de Estado da Saúde de São Paulo, São Paulo, Brasil; 2 Departamento de Biologia Animal, Universidade Estadual de Campinas, Campinas, Brasil; Lancaster University, United Kingdom

## Abstract

**Background:**

The scarcity of information on the immature stages of sand flies and their preferred breeding sites has resulted in the focus of vectorial control on the adult stage using residual insecticide house-spraying. This strategy, along with the treatment of human cases and the euthanasia of infected dogs, has proven inefficient and visceral leishmaniasis continues to expand in Brazil. Identifying the breeding sites of sand flies is essential to the understanding of the vector's population dynamic and could be used to develop novel control strategies.

**Methodology/Principal finding:**

In the present study, an intensive search for the breeding sites of *Lutzomyia longipalpis* was conducted in urban and peri-urban areas of two municipalities, Promissão and Dracena, which are endemic for visceral leishmaniasis in São Paulo State, Brazil. During an exploratory period, a total of 962 soil emergence traps were used to investigate possible peridomiciliary breeding site microhabitats such as: leaf litter under tree, chicken sheds, other animal sheds and uncovered debris. A total of 160 sand flies were collected and 148 (92.5%) were *L. longipalpis*. In Promissão the proportion of chicken sheds positive was significantly higher than in leaf litter under trees. Chicken shed microhabitats presented the highest density of *L. longipalpis* in both municipalities: 17.29 and 5.71 individuals per square meter sampled in Promissão and Dracena respectively. A contagious spatial distribution pattern of *L. longipalpis* was identified in the emergence traps located in the chicken sheds.

**Conclusion:**

The results indicate that chicken sheds are the preferential breeding site for *L. longipalpis* in the present study areas. Thus, control measures targeting the immature stages in chicken sheds could have a great effect on reducing the number of adult flies and consequently the transmission rate of *Leishmania (Leishmania) infantum chagasi*.

## Author Summary

Sand flies are the vectors of a number of pathogens that infect man, especially cutaneous and visceral leishmaniasis. The control of sand flies through residual house-spraying insecticide has proven inefficient in preventing the spread of these diseases in several areas of the globe. Sand flies have a life cycle including eggs, larvae and pupae that are found in the ground, and winged adults. We developed a soil emergence trap that allowed us to identify chicken sheds as the preferred breeding sites for *L. longipalpis* that is the vector of visceral leishmaniasis in urban areas of municipalities in South-eastern region of Brazil. This finding opens up the possibility of controlling the immature stages which could complement the usual control strategies that focus on the adult fly.

## Introduction

Members of the *Lutzomyia longipalpis* (Lutz & Neiva) species complex are the main vector of *Leishmania (Leishmania) infantum chagasi* (Cunha & Chagas), the causative agent of the visceral leishmaniasis (VL) in Brazil and Latin America [1]–[3]. Its recent introduction and adaptation to domiciliary habitats in the urban and peri-urban areas of municipalities in every region of Brazil, including São Paulo state, has resulted in higher incidences of human and/or canine visceral leishmaniasis [4]–[8]. From 1995 to 2010, Brazil recorded 53,633 new cases of the disease, with an annual mean of 3,352 new cases [9]. From 1999 to 2011, São Paulo state recorded 1,927 new cases of the disease, with 169 deaths [10].

In Brazil, the VL control program focuses on the treatment of human cases, the euthanasia of seropositive infected dogs and insect vector control [5], [11], [12]. Since this disease is zoonotic, treatment of human cases does not affect transmission. The incidence of human infections seems to be associated with the number of infected dogs and vectors [13]–[15]. Culling dogs is unpopular and its impact on the reduction of the incidence of human VL is contradictory [12]–[14], [16]–[18]. Vectorial control is focused on the sand fly's adult stage through residual house-spraying insecticide [1], [5], [11], [12], [19]. Insecticide application is generally accepted by residents in affected areas, is more practical and, in theory, more effective than the other methods at controlling transmission [13]. However, due to the irregularity and low coverage of application, possible repellent action of insecticide, high cost when used on a large scale and its short residual effect this method is considered inefficient in the control of VL [12], [14], [19], [20].

In Brazil, the high abundance of *L. longipalpis* in conjunction with domestic animals, particularly dogs, which act as the amplification hosts for *L. (L). i. chagasi*, means that transmission occurs within relatively small areas represented by peridomiciles in rural and urban areas [19], [21]–[23]. In addition, dispersion studies have shown that the movements of *L. longipalpis* individuals are spatially focal [24], [25]. Such observations, coupled with the high frequency of males and females of different physiological age (empty, engorged and gravid) present in collections carried out in urban areas of several municipalities of São Paulo state (unpublished), indicate that the complete life cycle of *L. longipalpis* occurs in peridomicile habitats of these areas. Nevertheless, finding the preferred breeding microhabitat of the *L. longipalpis* and other sand flies species is a difficult task [26]–[28].

It is generally assumed that the immature stages of sand flies develop in shaded and moist terrestrial microhabitats, rich in organic nutrients, such as in or near leaf litter, bases of trees, animal burrows, animal sheds, and rock crevices [27], [29]–[32]. Nevertheless, there is no precise information on the preferred breeding sites of these important vectors. Among the techniques used to identify the natural breeding sites of sand flies, soil emergence traps are the preferred indirect method employed [26], [27], [29], [30], [33]–[37].

In this study, a new soil emergence trap design was used to identify the natural breeding sites of *L. longipalpis* in urban and peri-urban areas of two municipalities identified as endemic for VL in São Paulo state. A more detailed knowledge of the immature stages and their preferential breeding sites is essential to understand the vector's population dynamics and could be used to develop novel control strategies.

## Methods

### Study area

**Figure 1:**
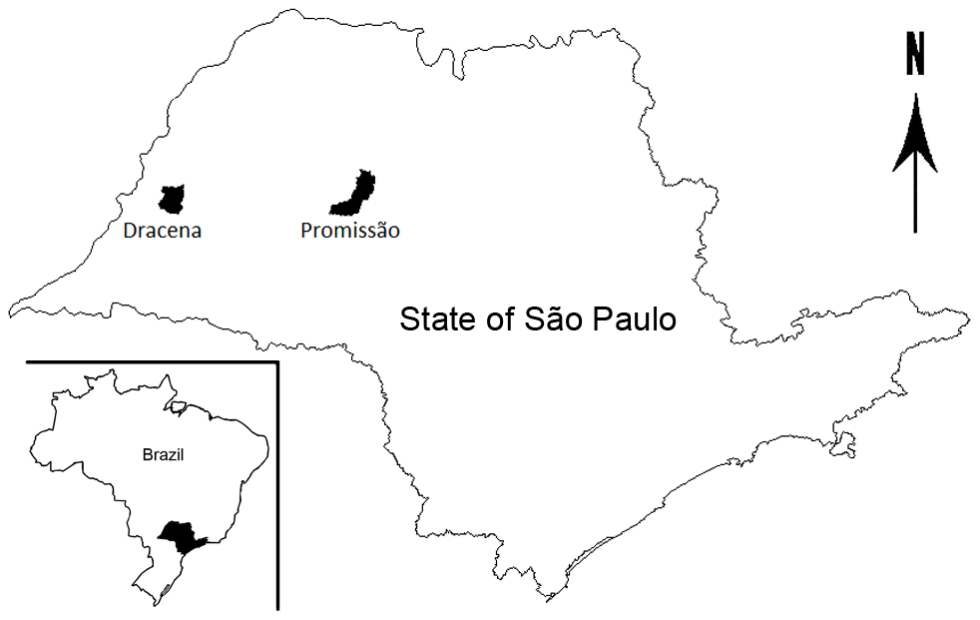
Localization of municipalities where breeding sites were investigated.

The municipality of Promissão (21°32′S 49°51′W) and Dracena (21°28′S 51°31′W) are located in the Western region of São Paulo state, Brazil (Fig. 1). Promissão has a total area of 787 km^2^ and approximately 37,570 inhabitants. Dracena has a total area of 489 km^2^ and approximately 41,000 inhabitants. According to Köeppen's climate classification, these areas are identified as Aw - Tropical wet and dry [38]. *Lutzomyia longipalpis* was first detected in Promissão and Dracena in 1999 and 2003, respectively. At the time of the present study, canine and human transmissions have been established in both municipalities. The houses in the urban and peri-urban areas of these municipalities usually have non-paved peridomicile with bushes and trees (e.g. fruit trees), and domestic animals shelters for dogs and chickens are common. In both municipalities, of the 20 blocks where the study was performed, chickens and dogs were present in approximately 10% and 30% of the dwellings respectively.

### Soil emergence traps

**Figure 2:**
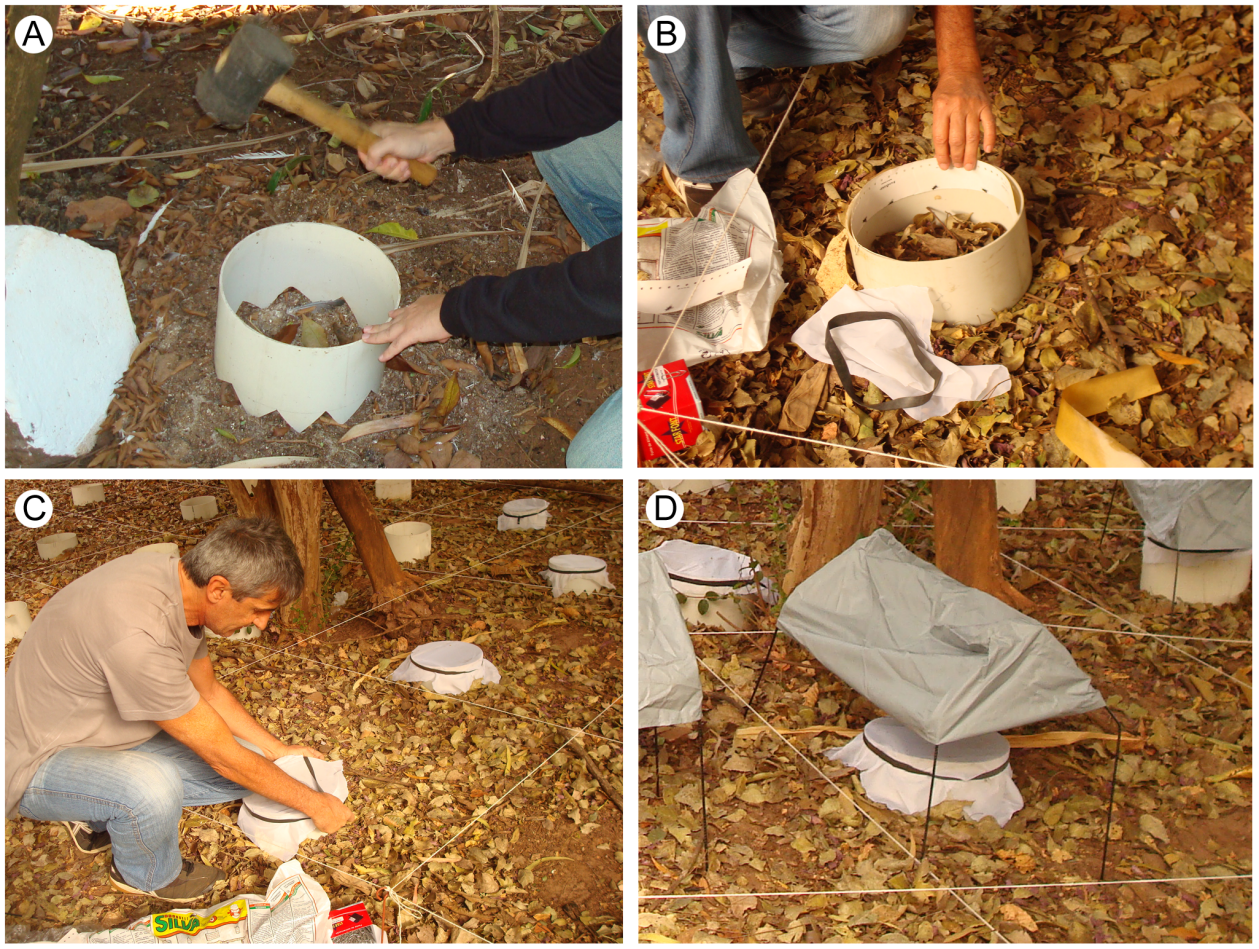
Emergence traps. **A**: Fixing emergence trap to the ground. The serrated bottom facilitates its fixation in the soil. **B**: Placing the adhesive paper. **C**: Covering the emergence trap with a sand fly-proof fine mesh to prevent sand flies escaping. **D**: Emergence traps covered with a small tent.

Two emergence trap designs were used in this study. The first one was that described by Casanova (2001) and a new one (described below) was developed by the same author (Fig. 2A–2D) through modifications in the polyvinyl chloride (PVC) pipe tube trap described by Ferro et al. (1997) [26], [35]. The emergence traps were 20 cm tall, with varying diameters of 10.16, 20.32, 25.40 and 44.60 cm, sampling 0.008, 0.032, 0.051 and 0.156 m^2^ of substrate respectively (the diameter reduced where necessary due to site conditions). The bottom edges of the PVC tubes were serrated to allow insertion at least 5 cm into the soil (Fig. 2A) of different microhabitats. After fixing it to the ground, the inner surface of the superior part of the tube was totally covered with a 4 cm wide adhesive paper (Fly – Catcher “The Stable”, Silva – made in Sweden), which was attached to the tube wall using paper clips (Fig. 2B). To prevent sand flies escaping, the top of the tube was covered with voile (a fine-mesh, fabric gauze) fixed with rubber bands (Fig. 2C). This way, the sand flies which emerge and fly inside the PVC tube end up landing on the adhesive paper, which works as a sticky trap. Finally, to protect the trap from the sun and rain, a small tent was placed over them, taking care to leave approximately 5 cm clearance between the trap and the tent walls (Fig. 2D).

### Sampling

Two series of continuous soil emergence trap collections were carried out from March 2005 to February 2006 and from September 2006 to January 2007 in the urban and periurban area of the municipalities. The first period was exploratory and its main objective was to identify possible *L. longipalpis* breeding sites and the fauna associated with them. The second period aimed to evaluate the spatial distribution pattern of immature stages in the two principal microhabitats positive to *L. longipalpis* in Promissão, during the first study period.

#### First series of collections

**Figure 3:**
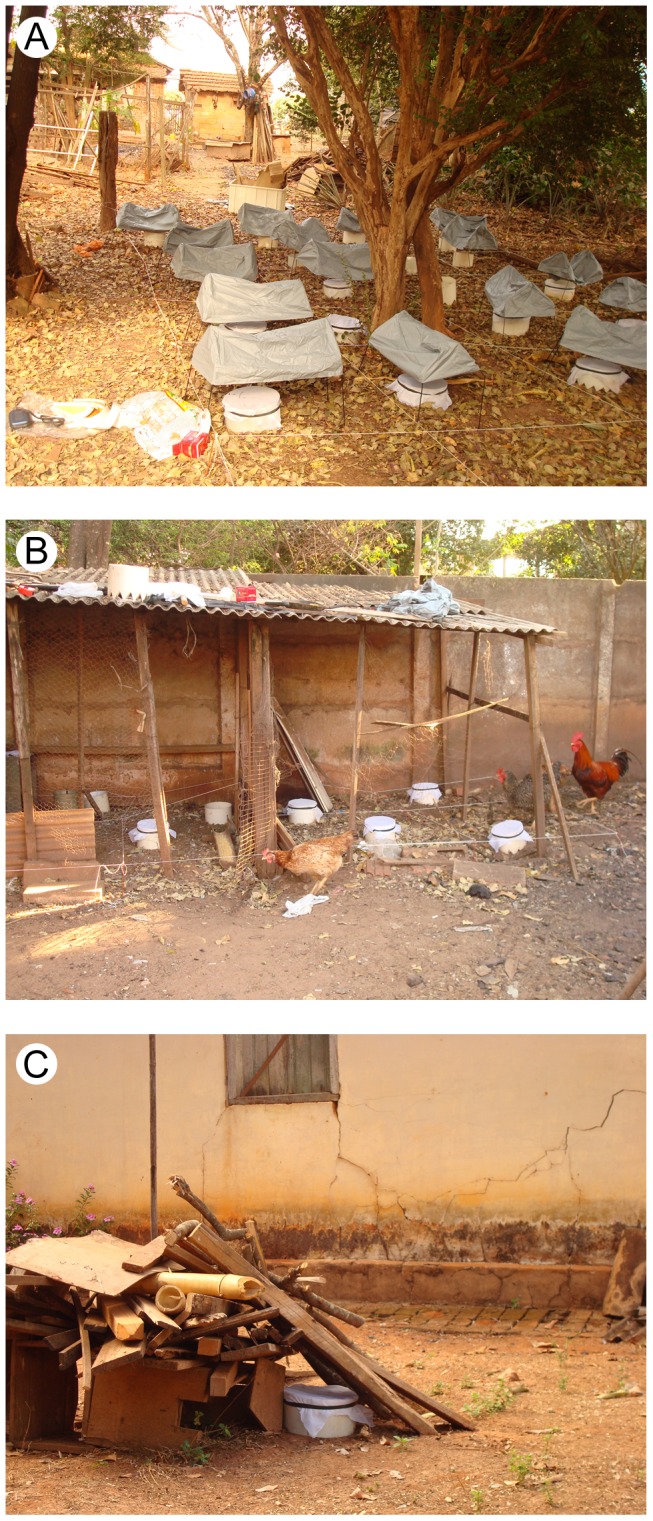
Examples of microhabitats. **A**: Leaf-litter microhabitat. **B**: Chicken shed microhabitat. **C**: Uncovered debris microhabitat.

Five to 13 emergence traps of different sizes were set in different microhabitats of 21 and 18 peridomiciles in Promissão and Dracena respectively, for at least 84 consecutive days. In the same microhabitat, the distance between the emergence traps varied, but they were set a minimum of 40 cm away from each other. All owners had given permission for the study to be conducted on their land. The dwellings were chosen according to both the presence of adult *L. longipalpis* in previous collections using CDC automatic light traps, and to the environmental characteristics that indicate the presence of probable sand fly breeding sites: non-paved and shaded peridomicile with organic material from animal or vegetables in decomposition stage. The main investigated microhabitats in this exploratory period were: a) leaf litter under tree (Fig. 3A); b) chicken shed (including chickens roosted in open-fronted roost adjoining brick walls and trees) (Fig. 3B); c) other animal sheds (pig, duck, dog and rabbit); and d) uncovered debris (pile of dry branch and logs of trees and leftover construction material) (Fig. 3C). Considering a mean time of 40 days for development of immature stages of *L. longipalpis* under laboratory conditions (data not published) the emergence traps were maintained in the same microhabitat point for 42 days (one cycle). After this period, the emergence traps were relocated to another point (about 10 cm) of the same microhabitat for a new 42-day cycle and were considered as new traps, because they sampled another area adjacent to that one in the previous cycle in the same site. The adhesive papers were removed and examined under a stereomicroscope for the presence of sand flies every 21 days. The flies captured on the adhesive papers were carefully removed with a brush soaked in xylene and kept for two hours in a vial with xylene to remove the glue. All sand flies were identified according to Galati (2003) [39].

To obtain preliminary information about the fauna associated with the breeding sites, soil samples (0.09 m^2^ and 5 cm deep) were taken with a hoe, adjacent to the emergence traps positive for *L. longipalpis* in Promissão. These samples were transferred to plastic bags and taken to the laboratory. The immature and adult stages of arthropods were separated in Macfayden's Funnel [30], maintained in ethanol (70%) and identified to Order or Family taxa, when possible. Data on the arthropods found on the adhesive papers of emergence trap positive for *L. longipalpis* was also assessed.

In order to monitor the adult population in the study areas of both municipalities, automatic CDC light traps were used weekly from 6 p.m. to 8 a.m. in the intradomicile and peridomicile habitats of 20 dwellings located, preferentially, on the blocks where the emergence traps were set. The distance between the CDC light traps and the emergence traps varied from five to 50 m, depending on whether the CDC light trap was in the same peridomicile or another in the same block and was influenced mainly by the relative size of the peridomiciles. The traps were installed near domestic animal shelters, preferably in chicken sheds. The monthly distribution of sand flies collected in CDC light traps and emergence trap was analyzed relative to the 10-day soil water balance [40]. Climate related data was provided by CIIAGRO (2012) and the Agricultural Climatology Department of Campinas Agronomical Institute [41].

#### Second series of collections

From September 2006 to January 2007, soil emergence traps were set in two chicken sheds and two leaf litter under tree microhabitats of one dwelling in Promissão. This dwelling was selected for its relatively high *L. longipalpis* production and high species diversity during the first series of emergence trap collection. A string grid was built over those microhabitats to divide the sampling area into 1×1 m (1 m^2^) quadrats (Fig. 3A). A 20.32 cm diameter (0.032 m^2^) emergence trap was set at random in each of the 24 quadrats of the first chicken shed and in the 15 quadrats of the second. Others 24 emergence trap, with a 25.40 cm diameter (0.051 m^2^), was also set in each quadrat of the two leaf litter under tree microhabitats. In contrast to the first period, in this part of the study emergence traps were maintained in the same point for 63-day periods.

### Statistical methods

Chi-square analysis, used to compare the proportion of positive microhabitats for *L. longipalpis* in each municipality, and Pearson correlation coefficient, calculated to examine the relationship between the number of *L. longipalpis* collected in emergence and CDC traps, were carried out using BioEstat (version 5.0; Mamirauá/CNPq, Belém, PA, Brazil). To evaluate the spatial distribution pattern of the immature stage, the variance-to-mean ratio (*s^2^*/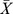), with chi-square statistic test to determining significantly departure from randomness χ^2^ = *SS*/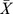 where *s^2^* is the variance and 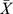 is the sampling mean and *SS* is the sum of squares) and the Morisita's Index (*I_d_ = nΣ x^2^−N/N (N−1)*, with chi-square statistic test to determine significant departure from randomness χ^2^ = *(nΣ x^2^/N)−N*, where *n* = number of samples; N is the total number of individuals collected, and *Σ* x^2^ is the squares of the number of individuals per sample, summed over all samples) were used [42]. In these indexes, values equal the unity indicate a random disposition, values smaller than the unity show a regular or uniform distribution, and values significantly higher than one indicate an aggregated or contagious disposition. The frequency of sand flies in CDC traps was obtained by month using William's geometric mean [43].

## Results

### First period of study

#### Emergence traps in Promissão

from March 2005 to February 2006, the leaf litter under tree microhabitat was investigated in all the 21 dwellings (61 sites), the chicken shed microhabitat was investigated in 15 (21 sites), the debris microhabitat in 13 (15 sites), and other animal sheds microhabitats in five (three rabbit hutches, one duck shed, and one pigsty). The relative low number of other animal shelters was due to the scarcity of other kinds of animals in peridomicile, excepting dogs. Although dogs were fairly common (there were dogs in about 30% of the dwelling in the study areas), their main function is usually to guard the dwelling. As such, the dogs were usually free in the peridomicile, sleeping in porches or utility areas whose floor are often concreted and cannot act as breeding sites.

A total of 386 emergence traps were used to sampled 26.92 m^2^ of soil: 15.62 m^2^ in leaf litter and base of tree trunks, 6.65 m^2^ in chicken sheds, 3.68 m^2^ in uncovered debris and 0.97 m^2^ in other animal sheds (pig, duck, and rabbit) (Table 1). Although the proportion of positives emergence traps for sand flies was low (5.95%), breeding sites were detected in approximately 40% of studied dwellings and all of them had one or more chicken sheds. Eleven (52.38%) chicken shed and four (6.56%) leaf litter under tree microhabitats were positive for sand flies species. The proportion of chicken sheds positive to *L. longipalpis* was significantly higher than in leaf litter under tree (χ^2^ = 20.39, df = 1, p<0.001) (Table 1).

**Table 1:** Summary of *Lutzomyia longipalpis* catches in soil emergence traps in the municipality of Promissão, from March/2005 to February/2006.

Microhabitat	No. sites (+sites*)	No. traps (+traps**)	Area*** (m^2^)	Male	Female	Flies/m^2^
Chicken shed	21 (11)	101 (19)	6.65	91	24	17.29
Leaf litter under tree	61 (4)	207 (4)	15.62	2	2	0.26
Uncovered debris	15 (0)	57 (0)	3.68	0	0	0
Others	5 (0)	21 (0)	0.97	0	0	0
Total	102 (15)	386 (23)	26.92	93	26	4.42

* +sites: positive sites.

** +traps: positive traps.

*** area sampled by emergence traps.

Of the 131 sand flies collected in emergence traps, 119 (90.84%) were *L. longipalpis*, six (4.58%) *Evandromyia cortelezzii* (Brèthes 1923), three (2.29%) *Evandromyia lenti* (Mangabeira 1938), two (1.53%) *Evandromyia termitophila* (Martins, Falcão & Silva 1964), and one (0.76%) *Nyssomyia neivai* (Pinto 1926). Except for four *L. longipalpis* collected in the leaf litter under tree microhabitat, all the other sand flies were collected in the chicken sheds microhabitat.

The *L. longipalpis* male/female ratio was 1∶1 in the leaf litter under tree microhabitat and 3.8∶1 in chicken shed (Table 1). The frequency distribution analysis showed that 70 (60.87%) *L. longipalpis* individuals were collected in only four (with 34; 13; 12 and 11 individuals in each trap) of the 19 positive emergence traps set in the chicken shed microhabitat. The number of sand flies collected by emergence traps fixed in chicken shed microhabitat varied from 0 to 34 individuals (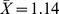, *SD* = 40.87) and no relation with the sampled area of traps was found (*r* = −0.180, *df* = 17, *p*>0.05).

**Figure 4:**
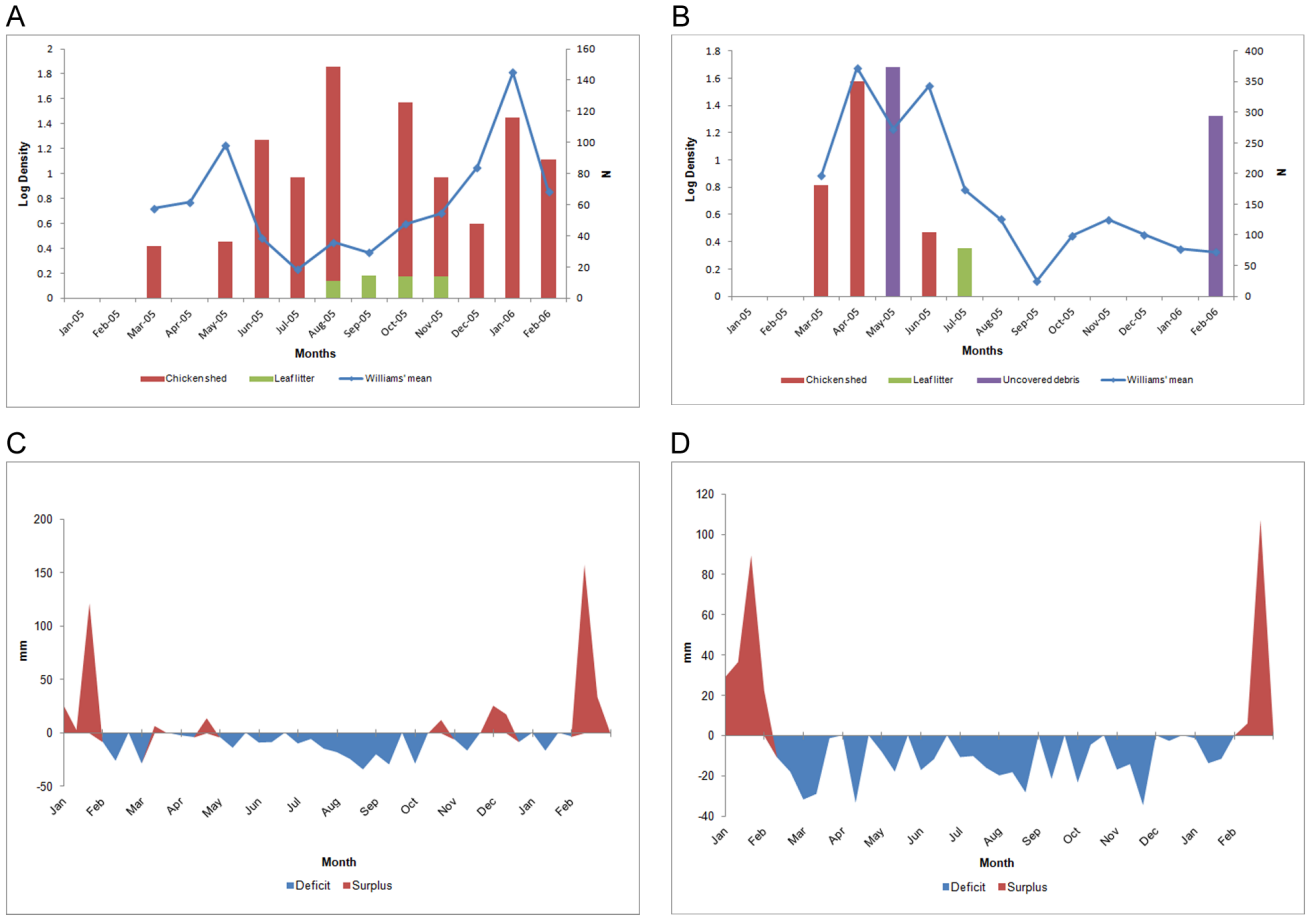
Distribution of *Lutzomyia longipalpis and* extract of the ten–day soil water balance. Monthly William's average (N) and log of density (fly/m^2^) of individuals collected in CDC and emergence traps, respectively, from March/2005 to February/2006. **A**: Promissão and **B**: Dracena. Water balance data from January/2005 to February/2006, for the municipality of **C**: Promissão and **D**: Dracena.

Considering that the number and size of the emergence traps in each microhabitat were not the same, the number of *L. longipalpis* collected in those traps was transformed into the number of individual per area of sampled microhabitat by the emergence traps (Table 1). Across all microhabitats, 4.42 *L. longipalpis* were collected per square meter (m^2^) sampled. For the chicken shed microhabitat, the total density was 17.29 individuals per m^2^, and for leaf litter under tree microhabitat only 0.26 per m^2^ (Table 1). *Lutzomyia longipalpis* was collected in the chicken shed microhabitat almost every month during the study period, but with a peak in August/2005 (Fig. 4A). This month corresponded to intense water deficit period as indicated by the 10-day soil water balance data (Fig. 4C).

#### Emergence traps in Dracena

the leaf litter under tree microhabitat was investigated in all the 18 dwellings (62 sites), the uncovered debris microhabitat in 10 (12 sites) of them, the chicken shed in eight (10 sites), and the kennel microhabitat in only two (two sites).

A total of 576 emergence traps were used to sampled 37.10 m^2^ of soil: 31.74 m^2^ in leaf litter and base of tree trunks, 2.45 m^2^ in chicken sheds, 2.25 m^2^ in uncovered debris and 0.66 m^2^ in kennel (Table 2). Only three (16.6%) peridomiciles and four (0.69%) emergence traps were positive for *L. longipalpis*: one chicken shed, one leaf litter and two uncovered debris microhabitats. Overall, the proportion of positive leaf litter microhabitat was significantly smaller than in uncovered debris and chicken shed microhabitats (χ^2^ = 58.53, df = 2, p<0.001) but no statistical difference was found between the proportion of positivity of the last two (χ^2^ = 0.065, df = 1, p>0.05).

**Table 2:** Summary of *Lutzomyia longipalpis* catches in soil emergence traps in the municipality of Dracena, from March/2005 to February/2006.

Microhabitat	No. sites (+sites*)	No. traps (+traps**)	Area*** (m^2^)	Male	Female	Flies/m^2^
Chicken shed	10 (1)	78 (3)	2.45	10	4	5.71
Leaf litter under tree	62 (1)	413 (1)	31.74	2	2	0.13
Uncovered debris	12 (2)	74 (2)	2.25	6	5	4.89
Kennel	2 (0)	11 (0)	0.66	0	0	0
Total	86 (4)	576 (6)	37.10	18	11	0.78

* +sites: positive sites.

** +traps: positive traps.

*** area sampled by emergence traps.

All the 29 sand flies collected in emergence traps in this municipality were *L. longipalpis*: 14 individuals were collected in chicken sheds (5.71 flies per square meter sampled), four in leaf litter (0.13/m^2^) and 11 in uncovered debris (4.89/m^2^). The *L. longipalpis* male/female ratio was 1∶1 in leaf litter under tree microhabitat, 2.5∶1 in chicken sheds, and 1.2∶1 in uncovered debris (Table 1). The frequency distribution analysis showed that 19 (65.51%) *L. longipalpis* individuals were collected in only two traps (one trap with 12 individuals in a chicken shed and one trap with seven individuals in uncovered debris). The number of sand flies collected by emergence traps fixed in all microhabitats varied from 0 to 12 individuals (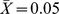, *SD* = 15.02) and no relation with the sampled area was found (*r* = −0.341, *df* = 4, *p*>0.05). *Lutzomyia longipalpis* was only collected in six months of the study period, with a peak in April and May/2005 (Fig. 4B). These months corresponded to moderate or intense water deficit periods kept by sporadic low rainfall index as indicated by the 10-day soil water balance data (Fig. 4D).

Neither in Promissão nor Dracena, were sand flies collected in soil with a high concentration of fresh chicken faeces, such as that found under roosts. The greatest number of sand flies were collected in the areas immediately surrounding the chicken sheds, where the organic matter such as chicken faeces and chicken food is completely degraded, drier, mixed in the soil and probably suitable as a food source for the larvae.

#### CDC collections

In the same study period, from March 2005 to February 2006, a total of 3,860 (3,070 males and 790 females) and 9,883 *L. longipalpis* (7,607 males and 2,276 females) were collected in CDC light traps set in the 20 dwellings in Promissão and Dracena, respectively. More than 85% of individuals (3,414 to Promissão and 8,763 to Dracena,) were collected in the peridomicile habitat, principally in CDC traps situated in the chicken shed microhabitat. *Lutzomyia longipalpis* was collected in every month in both municipalities, with higher abundance during May/2005 and January/2006 in Promissão (Fig. 4A) and during April, May and June in Dracena (Fig. 4B). These months corresponded to moderate water deficit periods kept by sporadic low rainfall index as indicated by the 10-day soil water balance extract (Fig. 4C, 4D).

In Promissão no significant correlation was found between the density of *L. longipalpis* observed in emergence traps in chicken sheds and CDC trap collections during the study months (*r* = 0.007, *df* = 10, *p*>0.05). On the other hand, this correlation was significantly positive to Dracena, when the density for the three positive microhabitats, chicken shed, leaf litter under tree and uncovered debris, is considered (r = 0.629, *df* = 10, *p*<0.05).

#### Associated fauna

In the same emergence traps where *L. longipalpis* were collected, the following taxonomic groups were collected: Hymenoptera (Formicidae), Coleoptera (adults), and Diptera (Sciaridae, Sphaeroceridae, Mycetophilidae, Phoridae, Fanniidae, Scenopinidae, Cloropidae, Caliphoridae, Lauxaniidae, Dolichopodidae, Milichiidae, Muscidae, Drosophilidae, Cecidomyidae, Sepsidae). In the soil samples obtained adjacent to emergence traps where *L. longipalpis* was present, adults or larvae from the following taxa were also collected: Psocoptera, Coleoptera, Hymenoptera, Homoptera, Araneidae, Collembola and Diptera (Drosophilidae, Sciaridae, Chironomidae, Phoridae, Sepsidae, and other Nematocera larvae). The Scenopinidae larvae are recognised predators of other Diptera larvae [44].

### Second period of study

#### Emergence traps in Promissão

In the second series of collections, from September 2005 to January 2007, a total of 16 *L. longipalpis* (nine males and seven females) were collected in soil emergence traps. Only one male was collected in one leaf litter under tree microhabitat. One male and one female were collected in chicken shed-2 and 13 individuals (seven males and six females) were collected in chicken shed-1. In this last case, five out of six positive traps were set in adjacent quadrats and the spatial distribution pattern of *L. longipalpis* immature stages evaluate by the variance mean ratio, 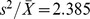 (χ^2^ = 169, df = 71, p<0.001) and the Morisita's Index, I_d_ = 9.230, χ^2^ = 169, df = 71, p<0.001) indicates a spatial contagious distribution.

### Discussion

The new design of emergence trap used in the present study is easy to construct, very straightforward to install and allows for longer periods between visits to the traps to remove sand flies (every 20 days, approximately).

Clearly, the chicken shed was the microhabitat that contributed the most to the high positivity of investigated dwellings, for the high proportion of positivity of emergence traps and to the increased number of *L. longipalpis* collected in the municipality of Promissão. In Dracena, chicken sheds were also the most productive microhabitat for *L. longipalpis*, however with a much lower number of positive emergence traps and sand flies than found in Promissão. Probably, this was due to both the smaller number of chicken shed investigated (about 50% smaller) and sampled area in the chicken shed microhabitat (about three times smaller) than that in Promissão.

Compared to the leaf litter under tree microhabitat, the chicken shed and uncovered debris areas are much smaller, and the population boundaries are readily apparent. This facilitates sampling of the population and estimating its size, particularly if the population shows contagious spatial distribution. In this sense, although in the present study the leaf litter under tree microhabitat has contributed only 5.40% of the total *L. longipalpis* collected, one should not ignore the possibility of a high production of *L. longipalpis* in this microhabitat, because this species is by far the most common in the peridomiciles of the studied areas. However, the differences in microhabitat size were most likely not the reason for the great difference observed between the positivity and productivity of these microhabitats. The chicken sheds are probably more favourable breeding sites because they are important blood feeding and resting sites for females of *L. longipalpis* and the abundance of faeces can provide a source of organic material for larval food.

One advantage of employing emergence traps to detect sand fly breeding sites is that they allow estimates of population densities from the observed productivity of breeding sites, expressed in adults/area/time [29], [45]. Considering the period of investigation, the number of the traps and the sampled area, the estimated production of *L. longipalpis* in the chicken shed microhabitat was by far the highest among all the researched microhabitats in the present study. For instance, the density of 17.29 sand flies per m^2^ or 41.17 sand flies per 100 m^2^ per day estimate for the total of chicken shed microhabitat in Promissão is a much higher value than found for *L. longipalpis* and other species in Neotropical region. In emergence traps set near pigsty microhabitats Ferro et al. (1997) estimated a density of 4.96 *L. longipalpis* per m^2^ sampled [26]. Considering other sand fly species, Rutledge & Ellenwood (1975) in Panama, and Arias & Freitas (1982), Casanova (2001) and Alencar (2007) in Brazil, estimated from the total of sand flies species captured in the litter of the forest floor, a production of 24.4, 4.1, 24.0 and 5.8 sand flies per 100 m^2^ per day, respectively [29], [33], [35], [36]. Notably, the 34 *L. longipalpis* collected in one emergence trap with 20.32 cm of diameter (0.032 m^2^), set in a chicken shed in Promissão, corresponded to a density of 1,062.5 sand flies per m^2^. Besides that, the high density of 50.7 sand flies per m^2^ obtained in chicken shed microhabitats during August 2005 in Promissão allowed to estimate a production of 121.7 *L. longipalpis* per 100 m^2^ per day. This high potential production of *L. longipalpis* agrees with the high abundance of adults in CDC traps set in the peridomicile.

The sexual ratio pro males may be a result of a possible greater male vagility after emergence facilitating their contact with the adhesive paper. Some of the male flies collected in the emergence traps still showed partial or no rotation of their genitalia. However, there is still the necessity to evaluate the efficiency of the traps of different dimensions to collect the emergence population inside them. This can be tested by releasing a known number of pupae or newly emerged adults into the traps, preferably at the central point [45].

Although the sampling of the microhabitats in the first period of study had been done with an uneven number of traps of different sizes, the intensive sampling, the concentration of a great number of *L. longipalpis* in few traps and the great number of traps with few or no flies seemed to be a strong indication of its contagious spatial distribution pattern. During the second study period, designed to identify where the concentrations occurred, and how great and how frequent they were, the indices used also showed a contagious distribution pattern. However, the abundance of *L. longipalpis* was low and new long term experiments, with greater number of traps and an exact positioning in the grid are necessary to allow the use of more accurate methods. These results suggest that females lay their eggs in clutches in restricted microhabitats, as indicated by Ferro et al. (1997) [26]. Probably the female sand fly in this, and other, species has the ability to detect food, shade, humidity and physical-chemical soil constitution, which are generally graded in a habitat. Some studies have shown that female sand flies locate an appropriate site by orienting towards semiochemical oviposition attractant components of eggs and animal faeces [46]. Contagious spatial distribution is the pattern commonly reported for insects in natural environments [45] and has already been suggested for sand flies species in forest floor microhabitats [29]. The contagious spatial distribution pattern can be responsible for the commonly reported difficulty in finding immature forms in soil samples or adult forms in emergence traps [27], [28].

The associated fauna may offer important information on species which might act as a sand fly breeding site indicator. Detecting larvae from a Diptera predator group in the same chicken shed sample where *L. longipalpis* were found is interesting from the biological control perspective. Collecting *E. cortelezzii*, *E. lenti*, *E. termithophila* and *N. neivai* in chicken shed together with *L. longipalpis* also points to the importance that this environment can have in the ecoepidemiology of cutaneous leishmaniasis.

The high abundance of *L. longipalpis* detected in the CDC traps fixed in peridomicile associated with animal pens, especially chicken sheds, has been frequently observed in several other rural and urban areas of Brazil [21], [47]–[49]. The lack of correlation between the number of adult sand flies collected in CDC traps and the number of immature stages in soil samples, or adults in emergence traps, is not uncommon [26], [50], [51]. This could be due to an accumulation of multiple generations of flies in the CDC trap, in addition to the fact that adults are attracted to the host from an area considerable larger than the emergence microhabitats that are sampled. Probably the combined effects of rainfall, air temperature and evapotranspiration, which determine the soil water balance, influence the quality of the breeding site microhabitats and consequently determine the adult sand fly population fluctuations [26], [29], [48], [52], [53]. In the present study, moderate water deficit periods seem to be favorable for population increase and periods of high water surplus seem to negatively affect the immature and adult populations of *L. longipalpis*. For Promissão, the rising curve for adults from October on, which led to a peak in December, may have been influenced by moderated water surplus periods between October and December, which only happened in this municipality and not in Dracena. On the other hand, longer periods of intense draught, like the one which happened more evidently in Dracena (water balance deficit from March to December), may be detrimental to the immature stages. The same has been observed with *L. longipalpis* and other sand fly species in urban and rural areas of São Paulo state [48], [53].

Both the high proportion of positive sites and high density of individuals in emergence traps indicate that the chicken shed microhabitat is the preferential breeding site of *L. longipalpis* in the study areas. In this sense, a new item – act as breeding sites to *L. longipalpis* - can be added to list of Alexander et al (2002) as factor associated to chicken sheds that increases the risk of transmission of *L. (L.) i. chagasi* to humans in Brazil [22]. Raising chickens is relatively common and both culturally and socio-economically important in urban, peri-urban and rural areas of the municipalities in all regions of Brazil. Its importance in the ecoepidemiology of zoonotic visceral involves some type of balance between zooprophylaxis, maintenance of sand fly populations and attraction of reservoir hosts [22]. Spatial analysis studies to detect areas at increased risk for visceral leishmaniasis such as those developed by Cerbino-Neto et al (2009), could evaluate the presence of chicken in the neighbourhood as an environmental factor associated with incidence of the disease [54]. It would be of interest to evaluate the efficiency of vector control interventions, such as environmental change and insecticide (chemical or biological), in chicken shed microhabitats, with the aim of reducing the adult population. The abundance of the female population size is a critical parameter of vectorial capacity [53], [55].

If the chicken shed, as a preferential breeding site, has an important role in determining the population abundance of adult forms of *L. longipalpis*, control strategies aimed at immature stage population in this microhabitat could be cheap, practical, and effective at reducing the populational abundance of females and consequently, the transmission rate. Furthermore, the efficacy of residual insecticide and others potential control strategies against adult forms - i.e. impregnated dog-collar [17], [56], zooprophylaxis [22] synthetic sand fly pheromone in conjunction whit insecticide [47], [57] – could be improved through of the control of immature stages of *L. longipalpis* population in chicken shed microhabitats.
